# Book Review

**Published:** 1940

**Authors:** 


### Reviews of Books

**Pioneers in Abdominal Surgery.** By Z. Cope, M.D., M.S., F.R.C.S. Pp. xii., 135. Illustrated. London: Oxford University Press (Humphrey Milford). 1939. Price 7s. 6d.—This is a very interesting little essay in the history of surgery. The author passes in review each of the standard operations for acute abdominal conditions, beginning with intestinal obstruction and passing on to appendicitis, perforated gastric ulcer, and ruptured ectopic pregnancy, and describes the first recorded operation for each. A valuable feature is the excellent series of photographs, thirty-one in all, of the pioneers, derived from many countries. Naturally, Great Britain is well represented.

**Physics for Medical Students.** Second Edition. By J. S. Rogers, B.A., M.Sc., F.Inst.P. Pp. xiii., 304. Illustrated. Melbourne: Melbourne University Press. 1939. Price 12s. 6d.—This book should prove of great interest to all members of the medical profession who wish to extend their acquaintance with the physical discoveries and principles underlying some of the recent advances in medicine. It is also to be recommended to physicists, in particular to those engaged in the teaching of medical students, as a clear exposition of those branches of their science which are applied in medicine, together with a brief account of the applications from a physicist's standpoint. As a text-book for medical students in this country, it is too advanced, since it assumes a knowledge of elementary physics approaching that of the 1st M.B. standard. The better student, however, should find it a stimulating book to read in conjunction with a more elementary one.

**A Synopsis of Surgical Anatomy.** Fourth Edition. By A. L. McGregor, M.Ch., F.R.C.S. Pp. xvii., 664. Illustrated. Bristol: John Wright & Sons Ltd. 1939. Price 17s. 6d.—The fact that this book has reached its fourth edition in the seven years which have elapsed since its first appearance should be sufficient evidence that it fills a real need. A book of nearly seven hundred pages, crammed with facts from cover to cover, can scarcely be regarded as a student's text-book for systematic study, for the average student does not like Applied Anatomy, nor does he appreciate its value and importance. This book, then, must find its chief function as work of reference or for occasional consultation. In such a capacity it could hardly be found wanting, for the range of the information contained in it is vast, the facts are presented in a simple and systematic way, and few omissions or statements open to challenge are to be found. It is curious, perhaps, to find no mention of strangulated hernia, nor of that great life-saving measure, Fowler's position, and its anatomical explanation. A brief mention of the problems connected with ascites, and the operations designed for its relief or cure might well be included. A word of warning might be given as to where the ureter is to be found after the peritoneum has been stripped back, for it sticks to the peritoneum and does not lie in the extra-peritoneal fat overlying the iliac vessels. We have seen inexperienced operators seeking it here in vain. It is interesting to see a reference to a case which seems to support the possibility of an occurrence which some might rate impossible, namely the escape of an ectopic testis down the femoral canal. Altogether a most stimulating book and full of interest : a search of its pages can hardly fail to be helpful to one in difficulty over a problem of Anatomy, whether commonplace or obscure. The new edition should enhance a reputation already earned by its predecessors.

**Blood Transfusion.** By V. H. Riddell, M.D., F.R.C.S. Pp. xiv., 370. Illustrated. London : Oxford University Press (Humphrey Milford). 1939. Price 21s.—This book is the first publication which reviews the subject of Blood Transfusion since 1922. During this time an immense bibliography has collected, and the long list of references at the end of each chapter shows the wealth of material that has been consulted. Two-thirds of the book is devoted to an account of the author's personal experience, and where alternative methods in the practical aspect of blood grouping and transfusion exist, the most practical procedure is described to the exclusion of the rest. Inevitably this will lead to the personal criticism of certain methods of technique. In this case, however, the procedures have been so simplified, the methods so fully described and every step so beautifully illustrated, that even those experienced in the art of blood transfusion will acquire many new points of view which will be of ultimate practical value. No aspect of the indications, complications, dosage and rate of administration is omitted, and the old standing custom of transfusing a patient with the empirical quantity of one pint is finally relegated to the past. The latter part of the book discusses the modern methods of technique available for collecting and delivering blood. The therapeutic value of whole, citrated and stored blood is considered, except that the subject of stored blood is of necessity incomplete. This is undoubtedly the most complete work available, and it can be confidently recommended to all members and students of the profession, and especially to those physicians and surgeons who are personally concerned in the performance of blood transfusion.

**Common Skin Diseases.** Fifth Edition. By A. C. ROXBURGH, M.A., M.D., B.CH., F.R.C.P. Pp. xxxi., 416. Illustrated. London: H. K. Lewis & Co. Ltd. 1939. Price 15s.—"Good wine needs no bush," and little recommendation is needed for a text-book which has reached its fifth edition in seven years. The present edition includes several new illustrations in addition to details of methods of treatment which have come into general use since the last edition, one of the most notable being the use of emulsifying bases. X-ray dosage is now given in *r*-units as well as in pastilles. *Common Skin Diseases* has reached the established place which was predicted for it in 1932, and is probably the most popular smaller work on dermatology in use at the present time. It can be recommended without any hesitation to both students and practitioners.

**The Psychological Aspect of Delinquency.** Edited by G. de M. Rudolf, M.R.C.P., D.P.M., D.P.H. Pp. 64. London: Baillière, Tindall and Cox. 1939. Price 2s. 6d.—In 1938 the Bath and Bristol Mental Health Society arranged a course of lectures on the Psychological Aspect of Delinquency, and these are now reproduced in pamphlet form. Each of the six talks was given by a different lecturer, three of them Bristol doctors. The address by the Director of the Tavistock Clinic on "the Magistrate and Delinquency" is outstanding. The booklet suffers from a lack of cohesion, the treatment of the subject is elementary and much is left unsaid. In fairness to the organizers of the course it should be pointed out that each talk was followed by an hour's discussion, when many problems receiving scant attention in the lecture could be developed. Each address by itself is quite readable, but the absence of an index makes cross-references difficult. This book would be of interest to anyone wanting to obtain a superficial knowledge of the subject, and it may well prove thought-provoking to those who are accustomed to the older methods of dealing with delinquents.

**Massage and Remedial Exercises.** Fourth Edition. By NOEL M. TIDY, C.S.M.M.G. Pp. xii., 458. Illustrated. Bristol: John Wright & Sons Ltd. 1939. Price 15s.—Some books are positively inviting to read, some only to peruse, while others are frankly for reference. Tidy's *Massage and Exercises*, now in its fourth edition, comes under the third category. It is too well known among students of remedial exercises to need any recommendation. At first sight the volume is bewildering; there is so much crowded into it, the type and illustrations being on the small size. For those for whom it is written, namely students of the C.S.M.M.G., it is invaluable as giving the physical exercises for every condition in which that treatment can be of any use. Notes of other physical treatments, such as electricity and light, are added where applicable. A page on gas poisoning brings the book right up to date.

**Physical Signs in Clinical Surgery.** Seventh Edition. By HAMILTON BAILEY, F.R.C.S. Pp. xii., 310. Illustrated. Bristol, John Wright & Sons Ltd. 1940. Price 21s.—There is no disparagement meant to the text of this book, when we call attention to the great value of the very numerous illustrations. To glance through these is by itself an excellent education in clinical surgery. In the present edition more and better pictures have been introduced, and another new feature is a series of footnotes explaining who the various surgeons are (or were), whose names have been given to signs or diseases. Altogether an excellent book, both for students and for doctors. It is very well printed and published, as one would expect from the famous house that has turned it out.

**Synopsis of Surgery.** Eleventh Edition. By E. W. HEY GROVES, M.S., M.D., B.Sc., F.R.C.S. Pp. viii, 714. Illustrated. Bristol: John Wright & Sons Ltd. 1940. Price 17s. 6d.—All that is necessary in reviewing a book so long and deservedly popular is to indicate the improvements shown in this new edition. These are valuable. The treatment of fractures has been revised, and brought into line with modern specialist

ARMY MEDICAL LIBRARY
365589
WASHINGTON, D. C. U. S. A.

methods, and in particular with those associated with the name of Bohler of Vienna. Another greatly improved section is that dealing with the operative treatment of cleft palate, where the older methods have been replaced by those of Veau, Gillies, Wardill, or Denis Browne. The Bristol School of Medicine and Bristol printing and publishing have both enhanced their reputation by the success of this book.

**The Abdominal Injuries of Warfare.** By G. GORDON-TAYLOR, O.B.E., F.R.C.S., F.R.A.C.S. Pp. vii., 87. Illustrated. Bristol: John Wright & Sons Ltd. 1939. Price 10s. 6d. —This book might well be read with advantage by all surgeons likely to have to deal with abdominal war wounds. Those who have dealt with these conditions know full well that there are many important details in the operative technique which do not arise in ordinary abdominal surgery. The author has not set out to write a treatise on the subject, but offers, as he says, the book as a *companion guide* to the illustrations. These illustrate, well and clearly, many important points in surgery of this kind, and the text has much excellent advice to offer as to the type of case it is wise to leave alone, those on which transfusion, which has improved on the results of the last war, should be done, and those in which there should be no delay in treating operatively. He also emphasizes the necessity of speed; the "surgical tortoise," as he rightly says, is not conducive to living results, and this without doubt will be confirmed by all surgeons who have had to deal with these cases in large numbers. Transfusion, thorough search of the gut from end to end, gentleness and speed in handling with the least possible exposure of the organs are the methods to be employed if the patient is to survive.

**Fractures and other Bone and Joint Injuries.** By R. WATSON-JONES, B.Sc., M.Ch., F.R.C.S. Pp. xii., 723. Illustrated. Edinburgh: E. & S. Livingstone. 1940. Price 50s.—Watson-Jones has produced a book in which the whole subject of fractures and allied bone and joint conditions is considered with great thoroughness and clarity. Free use of illustrations and X-rays has amplified the text and added to the interest. The book opens with ten excellent chapters on general questions connected with fractures and their treatment, chapter three on adhesions and joint stiffness being a particularly fine section. Mr. Watson-Jones recommends the use of a leather boot to be worn over the plaster cast in place of the usual walking iron. This would, of course, involve a great increase in expense. The chapter on fractures of the dorsolumbar vertebrae is beautifully classified and illustrated where the low dorsal and lumbar vertebrae are concerned, but unfortunately the more difficult upper dorsal region is passed over with only a short paragraph and no illustrations. The chapter on low back strain and sciatica is a very helpful inclusion. As the author truly says, there have been innumerable methods designed for introducing Smith-Peterson pins into a fractured neck of the femur. His method does not appear the best. Perforation of the femoral artery in using the Hey Groves apparatus for this purpose can only be accounted for by a lapse in the elementary precaution of feeling for the arterial pulsation, which in the abducted leg is always medial to the point overlying the centre of the femoral head. There is an occasional jarring note provided by an overstatement, as for instance when a rather unpleasant-looking guillotine amputation is shown in illustration 107 with the caption " A result of skeletal traction." No method of treatment is free from its tragedies, most of these occurring in incompetent hands. After making out a well argued case for traction through the lower third of the tibia, confusion is spread by nearly every illustration showing traction through the os calcis as is the more usual practice. This contribution to the literature on fractures has the great attribute of being freely and excellently illustrated and having a text which is clear and concise, and in which a logical reason is always given to satisfy the enquiring mind. This book will undoubtedly be widely and profitably read.

**Thomson and Miles' Manual of Surgery.** 2 vols. Ninth Edition. Edited by A. Miles, M.D., LL.D., F.R.C.S., and the late Sir D. Wilkie. Vol. 1 pp. 656, Vol. 2 pp. 715. Illustrated. London: Oxford University Press (Humphrey Milford). 1939. Price 42s.—Not much need be said to commend a book which has survived for thirty-five years, and is the recognized exponent of the great Edinburgh school of surgical theory and practice. This edition comes from the hand of no less than twenty-eight contributors, and was planned—almost his last public service to the profession—by the late Sir David Wilkie. The illustrations have been overhauled and greatly improved, and it is claimed that the book has been extensively altered and brought up to date, and in part re-written. Unfortunately, the revision of these older text-books is seldom thorough enough.

We doubt if a student would get much credit in an examination if all he had to say about the treatment of pneumococcal peritonitis was, as advised here, to "open the abdomen" (which is useless), to "drain" (which is pernicious) and to "combat septicaemia" (which is vague). The iodine-in-milk treatment for actinomycosis is not noticed, nor the salvarsan treatment for anthrax. A good, practically new, section is contributed by Norman Dott on neurological surgery, and well demonstrates the great progress that has been made in the diagnosis and treatment of injuries and diseases of the brain.

**The Early Diagnosis of the Acute Abdomen.** Eighth Edition. By Zachary Cope, M.D., M.S., F.R.C.S. Pp. xv., 257. Illustrated. London: Oxford University Press (Humphrey Milford). 1940. Price 10s. 6d.—The acute abdomen is one of the great opportunities that come the doctor's way, for on his prompt decision life often depends. To ensure a right diagnosis he should read, mark and inwardly digest the contents of this book. They represent the compressed knowledge of an eminently sound surgeon of ripe experience. The author writes in a good didactic style, avoids digression, and gives to each of the various types of emergency a well-proportioned space. Sprinkled throughout the book are helpful classifications of symptoms, comparative tables of differential diagnosis and first-rate diagrams which are as lucid and telling as any the reviewer has seen in surgical works. Constant reference to this handy and inexpensive volume would enable the resident to get far more value out of a hospital appointment, and would help the general practitioner in his vital, but less frequent contact with the acute abdomen.

**British Red Cross Society Notebook.** Second Edition. Edited by St. J. D. Buxton, M.B., B.S., F.R.C.S. Pp. iv., 100. Illustrated. London: Oxford University Press (Humphrey Milford). 1940. Price 2s. 6d.—This notebook is a welcome addition to the Library at this particular time. Its size is not unwieldy and at the same time gives plenty of space for note taking. The diagrams are clear and boldly drawn, three essentials being well portrayed: (*a*) The Gross Anatomy. (*b*) Common Types of Fracture and Dislocation. (*c*) The First Aid Treatment of Serious Hæmorrhage. The Anatomical diagrams show at a glance the subject portrayed and leave the student space for marking in the names of the various parts. Unlike the official first aid text-books, this treats the subject of circulation and hæmorrhage in its proper place—namely first. Students attending a course of lectures will find this book invaluable for systematic note taking, and for reference purposes.

**A Pocket Medical Dictionary.** Fourth Edition. By L. Oakes, S.R.N., D.N., and T. B. Davie, M.D., M.R.C.P. Pp. xx., 409. Illustrated. Edinburgh : E. and S. Livingstone. 1940. Price 3s. 6d.—For those who have occasion for a handy little volume of this type this can be thoroughly recommended. The information is concise and accurate, and spelling and definitions will serve those who require help in these directions.

**An Atlas of the Commoner Skin Diseases.** Second Edition. By HENRY C. G. SEMON, D.M., F.R.C.P. Pp. xii., 272. Illustrated. Bristol: John Wright & Sons, Ltd. 1940. Price 42s.—A second edition of this admirable atlas has just been published. The book has been brought up to date, and the colour of some of the illustrations has been in some instances improved. It is a most valuable and practical handbook well worth the price charged. Would it be presumptuous to suggest that *Pellagra* is really a not uncommon disease in the British Isles, and that it frequently goes unrecognized from unfamiliarity with its appearance ?

**An Index of Treatment.** Twelfth Edition. By Sir ROBERT HUTCHISON, Bart., M.D., LL.D., F.R.C.P., and REGINALD HILTON, M.D., F.R.C.P. Pp. xv., 996. Illustrated. Bristol: John Wright & Sons, Ltd. 1940. Price 42s.—The new edition (twelfth) of Hutchison's *Index of Treatment* is most welcome. Several of the articles have been entirely re-written and a few new ones introduced, with the result that the work has been brought thoroughly up to date. Wright's Index Series in Medicine and Surgery really do, as the publishers claim, "form a complete reference library for the busy practitioner."

**Ear Model.** Designed by A. J. WRIGHT, M.B., F.R.C.S. Illustrated. Bristol : John Wright & Sons, Ltd. 1939. Price 21s.—This ingenious model enables the student or practitioner to familiarize himself with the appearances of the normal and pathological tympanic membrane. It has been placed in charge of the Librarian and is available for use by any student or practitioner who brings his own auriscope.

